# Characterization and Analysis of the Functional Differences of the Two Eclosion Hormones in Regulating Molting in the White Shrimp *Litopenaeus vannamei*

**DOI:** 10.3390/ijms252312813

**Published:** 2024-11-28

**Authors:** Yunjiao Li, Zecheng Li, Hongmei Ran, Zihan Fan, Fan Yang, Hu Chen, Bo Zhou

**Affiliations:** 1 Fisheries Research Institute of Sichuan Academy of Agricultural Sciences, Yibin 644000, China; yunjiaoli@scsaas.cn; 2 Key Laboratory of Tropical Hydrobiology and Biotechnology of Hainan Province, Hainan Aquaculture Breeding Engineering Research Center, School of Marine Biology and Fisheries, School of Breeding and Multiplication (Sanya Institute of Breeding and Multiplication), Hainan University, Haikou 570228, China; lizecheng@hainanu.edu.cn (Z.L.);

**Keywords:** *Litopenaeus vannamei*, eclosion hormone, molting, bioinformatics

## Abstract

*Litopenaeus vannamei*, with an annual production of 5–6 million tons and a value of USD 50–60 billion, is a cornerstone of global aquaculture. However, molting-related losses of 5–20% significantly impact this industry, and the physiological mechanisms of molting remain unclear. This study aims to elucidate the role of eclosion hormone (EH) in molting regulation and enhances the understanding of molting physiology in *L. vannamei*. This study investigated the role of (EH) in *L. vannamei* molting regulation. Two *EH* cDNAs, *LvEH I* and *LvEH II*, were identified, and their expression patterns across tissues and seven molting stages (A, B, C, D0, D1, D2, and D3) were analyzed. *LvEH I* was predominantly expressed in the gill, epidermis, and eyestalk, while *LvEH II* was mainly expressed in the eyestalk and brain. *LvEH I* was highly expressed in the eyestalk, epidermis, and gills at the D2 and D3 stages of molting, whereas *LvEH II* was highly expressed in both the D2 (brain) and D3 (eyestalk) stages. RNA interference (RNAi) targeting *LvEH I* revealed its critical role in molting, as silencing *LvEH I* disrupted the expression of molting-regulation genes, *ETH*, *CCAP*, *CHH*, *EH II*, *CDA*, and bursicon (*Burs*), significantly delaying the molting process. These findings highlight both *LvEH I* and *LvEH II* as indispensable for normal molting in *L. vannamei* and provide a foundation for developing effective molting management strategies to reduce industry losses.

## 1. Introduction

The Pacific white shrimp *Litopenaeus vannamei* is a globally widely farmed and highly productive invertebrate species [1,2]. The significant advantages of *L. vannamei,* such as its high production per unit area, excellent adaptability, high meat yield, and efficient feed conversion rate, make it one of the most ideal aquaculture species worldwide [3]. Its annual production is over 5 million tons, with an output value of 50–60 billion USD [4]. Given its high production and output value, improving current aquaculture techniques for *L. vannamei* will provide substantial economic benefits. Understanding the physiological regulatory mechanisms is fundamental to advancing aquaculture technology. However, many physiological regulatory mechanisms closely related to the aquaculture of *L. vannamei*, such as molting regulation, remain unclear.

Molting is an essential, periodic, and ongoing physiological process in *L. vannamei* and is closely related to various aspects of aquaculture, such as growth and development, reproduction, disease resistance, and stress resistance [5,6,7,8]. Generally, the molt cycle of *L. vannamei* is divided into four stages: postmolt (A/B-stage), intermolt (C-stage), premolt (D-stage), and ecdysis (E-stage). Based on the morphological changes in the setogenesis development of the endopodite, the molting cycle can be further subdivided into eight stages [6,7,9]. These stages include early and late postmolt (A and B), intermolt (C), onset of premolt (D0), early, intermediate, and late premolt (D1, D2, and D3), and ecdysis (E). *L. vannamei* undergoes a complex molting process nearly 50 times throughout its lifetime, with each molt posing a risk of mortality due to stress, nutritional deficiencies, environmental issues, and other factors [7,10,11]. Research has shown that the mortality rate caused by poor molting management may exceed 50%, resulting in considerable economic losses [12]. Currently, several strategies, including nutrition, environmental regulation, and hormone injection, have been used to improve the survival rate of *L. vannamei* molting [7,13,14,15,16,17]. However, the unclear physiological mechanisms of molting have led to unpredictable results from management strategies. Therefore, understanding these mechanisms is crucial for developing effective molting management practices.

Currently, systematic and comprehensive studies on molting regulation in crustaceans are lacking, with most related research primarily focused on insects. The molting behavior in insects is a complex physiological process regulated by 20-hydroxyecdysone (20E) [18], a mechanism that has also been demonstrated in crustaceans [19]. However, in insects, the regulation of 20E is primarily driven by the positive regulation of prothoracicotropic hormone (PTTH), whereas in crustaceans, 20E is mainly negatively regulated by molt-inhibiting hormone (MIH) and crustacean hyperglycemic hormone (CHH). Despite the regulatory differences in the upstream control of 20E between insects and crustaceans, current evidence suggests that the downstream responses are nearly identical [20]. These responses primarily involve the coordinated action of eclosion hormone (EH), ecdysis-triggering hormone (ETH), corazonin (CRZ), and bursicon (Bur), which together orchestrate the physiological regulation of molting [20]. EH is a molting behavior trigger hormone that plays a key role in molting starting in the molting process of arthropods [21]. In arthropods, *EH* has highly conserved functions and is essential in the molting process. Studies have shown that the molting behavior of *EH*-deficient fruit flies and *Tribolium castaneum* is disrupted, which eventually leads to a large number of deaths before molting [22,23]. Currently, only a few reports have confirmed the direct function of EH in the regulation of crustacean molting. *Scylla paramamosain* injected with *EH* double-stranded RNA (dsRNA) also died because of the inability to molt [24]. In palaemonid shrimp, RNA interference (RNAi) with the expression of *EH* in *Exopalaemon carinicauda* delayed molting behavior [21]. However, the function of EH in the economically important species *L. vannamei* remains unclear. Investigating its role and its coordinated mechanisms in the molting process holds significant value as a reference for optimizing molting management in *L. vannamei*.

In this study, homologous searching with transcriptome data was used to identify the EH of *L. vannamei*, and molecular cloning was employed to obtain the coding sequence (CDS) of *EH*. The expression levels of *EH* in ten tissues were subsequently measured via reverse transcription–quantitative polymerase chain reaction (RT–qPCR) to identify the primary tissues where *EH* performs its biological functions. The expression levels of *EH* in these primary tissues were then assessed across seven molting stages to determine in which molting stage EH plays a significant physiological role. However, while we were preparing RNAi experiments for *EH*, other researchers had already published studies on *EH II* interference in *L. vannamei* [25]. Therefore, this study focused on investigating the function of *EH I* by RNAi and evaluating its effects on molting progression and the associated molting regulatory hormone systems. This study is conducive to understanding the molting physiology of *L. vannamei* and provides a theoretical basis for the formulation of scientific molting management procedures for *L. vannamei*.

## 2. Results

### 2.1. Identification of Two LvEHs from L. vannamei

**Figure 1:**
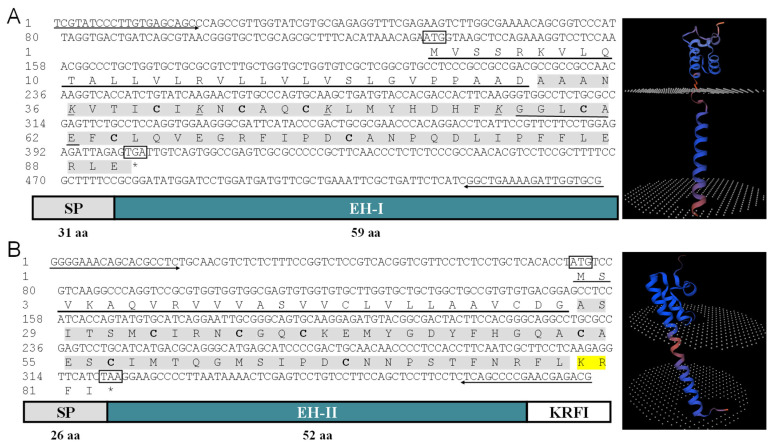
cDNA sequences, deduced amino acids, and three-dimensional structural characteristics of EH I (**A**) and EH II (**B**). The start and stop codons are marked with boxes, the primers are marked with arrows, the signal peptide is marked with bold black underlines, the mature peptide is marked with gray shading, and the structurally conserved cysteine residues are marked with bold. Yellow shading is used to mark the cleavage site. * indicates the termination signal.

Two *EH* cDNAs with obvious differences were obtained from *L. vannamei* (Figure 1). The gene information has been submitted to the NCBI database and obtained the accession numbers PQ583330 and PQ583331.The length of the *LvEH I* cDNA is 545 bp, and the coding sequence (CDS) is 273 bp, which encodes 90 amino acids (Figure 1A). The *LvEH II* cDNA is 388 bp in length, while its CDS spans 249 bp, encoding a protein of 82 amino acids (Figure 1B). Bioinformatics prediction revealed that both LvEH I and LvEH II are located outside of cells, with signal peptides and no transmembrane domains (Figure 1A,B). The relative molecular mass of LvEH I is approximately 9.915 kDa, and the theoretical isoelectric point (pI) is 7.60. LvEH II is slightly smaller, with a relative molecular mass of approximately 8.934 kDa and a theoretical isoelectric point (pI) of 8.16.

**Figure 2:**
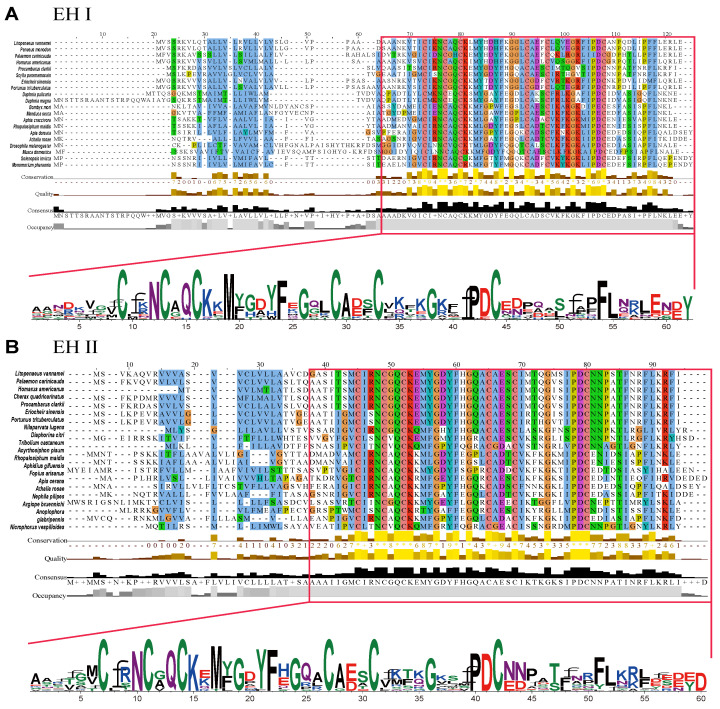
Multiple sequence alignment of EH I (**A**) and EH II (**B**).

**Figure 3:**
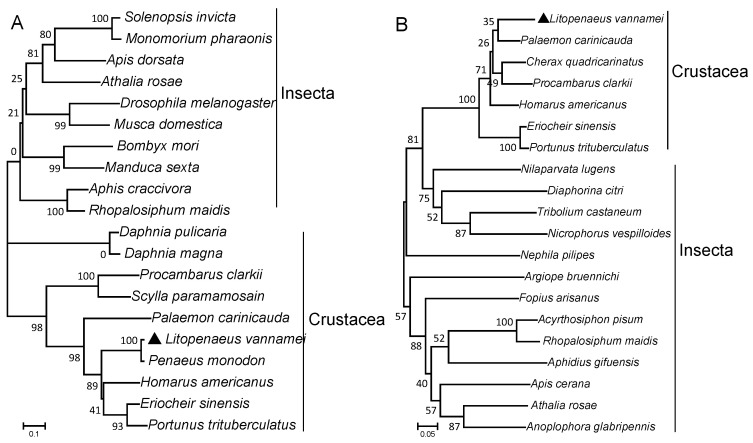
Phylogenetic tree of EH I (**A**) and EH II (**B**). The target sequences of this study are marked with black triangles.

LvEH I and LvEH II both contain a conserved eclosion superfamily domain, six highly conserved cysteine residues, and three corresponding disulfide bonds (Figure 1 and Figure 2). The eclosion superfamily domain in LvEH I ranges from 37 to 84 amino acids, and that in LvEH II ranges from 20 to 80 (Figure 1 and Figure 2). The predicted mature peptide of LvEH I contains six cysteine residues (Cys^9^, Cys^13^, Cys^16^, Cys^29^, Cys^33^, and Cys^44^) and three corresponding predicted disulfide bonds (Cys^9^-Cys^29^, Cys^13^-Cys^16^, and Cys^33^-Cys^44^) (Figure 1A and Figure 2A). LvEH II is similar to LvEH I but has slightly different specific sites. The six cysteine residues in the predicted LvEH II mature peptide are Cys^7^, Cy^s11^, Cys^14^, Cys^27^, Cys^31^, and Cys^42^, and the three disulfide bonds are as follows: Cys^7^-Cys^27^, Cys^11^-Cys^14^, and Cys^31^-Cys^42^ (Figure 1B and Figure 2B). The signal peptides of LvEH I and LvEH II are 31 amino acids (Met^1^-Asp^31^) and 26 amino acids (Met^1^-Gly^26^), respectively (Figure 1A,B). NeuroPred prediction revealed that amino acids 79–80 of LvEH II are the cleavage site of the neuropeptide (Figure 1B). The phylogenetic tree suggested that the LvEH I of *L. vannamei* is most closely related to that of *Penaeus monodon*, followed by that of *Homarus americanus* (Figure 3A). However, LvEH II of *L. vannamei* is most closely related to the sequence of *Palaemon carinicauda*, followed by *Homarus americanus* (Figure 3B).

### 2.2. Differential Expression of the Two EHs in Different Tissues

**Figure 4:**
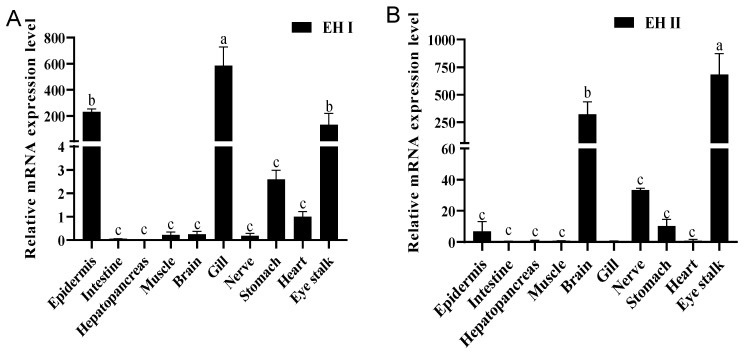
Relative expression of EH I (**A**) and EH II (**B**) in different tissues of *Litopenaeus vannamei*. The relative expression levels of EH I and II in 10 tissues were detected via qRT–PCR, and the statistics were calculated via the 2^−ΔΔCT^ method and are expressed as the means ± standard errors of the means (n ≥ 3). Lowercase letters indicate significant differences, with different letters showing a significant difference between groups.

To investigate the tissue-specific functions of the two *EH*s and assess potential functional differences, their expression levels were analyzed across 10 different tissues (Figure 4). *LvEH I* presented the highest expression in the gills, followed by the epidermis and eyestalks (Figure 4A). Although no significant differences were observed in the other seven tissues, *LvEH I* expression was detectable in the heart and stomach, whereas it was nearly absent in the remaining tissues (Figure 4A). In contrast, *LvEH II* was most highly expressed in the eyestalks, followed by the brain (Figure 4B), with weak expression observed in the nerves, stomach, and epidermis (Figure 4B). No detectable expression of *LvEH II* was detected in the intestine, hepatopancreas, muscle, gills, or heart (Figure 4B). The results of tissue expression suggested that *LvEH I* functions mainly in the eyestalks, gills, and epidermis, whereas *LvEH II* functions mainly in the eyestalks and brain; thus, these tissues were used for subsequent functional studies of EH.

### 2.3. Functional Differences Between LvEH I and LvEH II During Molting of L. vannamei

**Figure 5:**
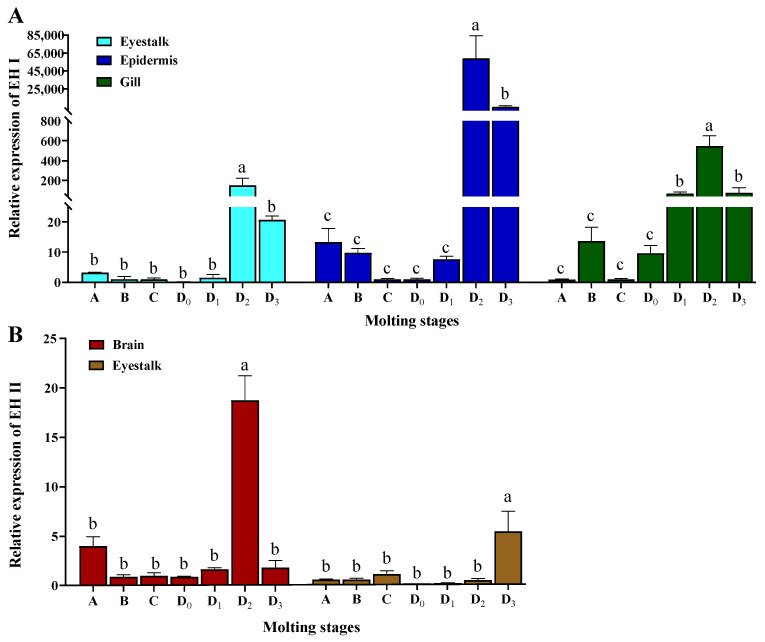
Expression of *EH I* (**A**) and *EH II* (**B**) in tissues with significantly higher expression during different stages of molting (n = 3). Lowercase letters indicate significant differences, with different letters showing a significant difference between groups.

According to the tissue distribution pattern of *LvEH*s, their expression levels at different molting stages (A, B, C, D0, D1, D2, and D3) in significantly highly expressed tissues were analyzed (Figure 5). For *LvEH I*, we focused on the eyestalk, epidermis, and gills, whereas *LvEH II* was examined mainly in the brain and eyestalk. As shown in Figure 5, both *LvEH I* and *LvEH II* were highly expressed in the premolting period (D stage), especially in the D2 stage. *LvEH I* was highly expressed in all three tissues at the D2 and D3 stages, whereas specific high expression was also detected in the gills at the D1 stage (Figure 5A). It is speculated that the action signal of *LvEH I* appears preferentially in the gills. In addition, there was no significant difference in *LvEH I* in various tissues at stages A, B, C, and D0. In contrast, *LvEH II* had the highest expression in the brain at stage D2 and the highest expression in the eyestalk at stage D3 (Figure 5B). There was no significant difference in the expression of *LvEH II* in the brain and eyestalks at stages A, B, C, D0, and D1 (Figure 5B).

### 2.4. Effects of RNAi with LvEH I on Molting Progression

**Figure 6:**
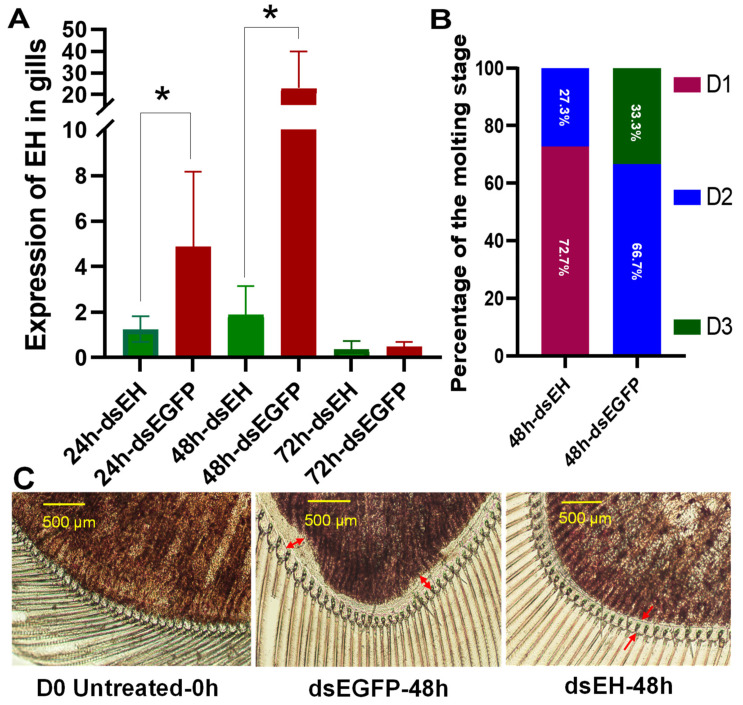
Effects of ds*EH I* injection on *EH I* expression in gills (**A**), molting progression (**B**), and molting microscopic characteristics (**C**). * represents significant differences, and *p* < 0.05. Arrows and bidirectional arrows are used to mark the space between the cuticle and epidermis.

During the course of our research, a study on the knockdown of *EH II* was published [25], prompting us to focus on the knockdown of *EH I* in this study. Given that *EH I* was highly expressed in the gills at the D1 stage (Figure 5A), dsRNA was administered at the D0 stage. We monitored the expression of *EH I* in the gills following ds*EH I* injection and found that the knockdown effect persisted for more than 48 h but diminished before 72 h (Figure 6A). Consequently, statistical analysis of molting progression within 48 h post ds*EH I* injection revealed a significant delay in molting. Specifically, only 27.3% of the shrimp advanced to the D2 stage, while 72.7% remained at the D1 stage. In contrast, 66.7% of the control group injected with ds*EGFP* reached the D2 stage, and 33.3% progressed to the D3 stage (Figure 6B). Morphological observations further confirmed this delay. In the ds*EH I*-silenced group, the degree of separation between the cuticle and epidermis corresponded to the D1 stage, while in the control group, it matched the D2 stage (Figure 6C). Specifically, prior to this experiment, the shrimps were in the D0 stage, marked by the initial separation of the cuticle and epidermis. In the ds*EGFP*-injected group, the shrimps progressed to the D2 stage, evident by an enlarged space between the cuticle and epidermis (highlighted by the red arrow). Conversely, in the ds*EH I* injection group, the shrimps remained in the D1 stage, as indicated by a red arrow pointing to the separation between the cuticle and epidermis (Figure 6C).

### 2.5. Effects of RNA Interference with LvEH I on Molting-Related Genes

**Figure 7:**
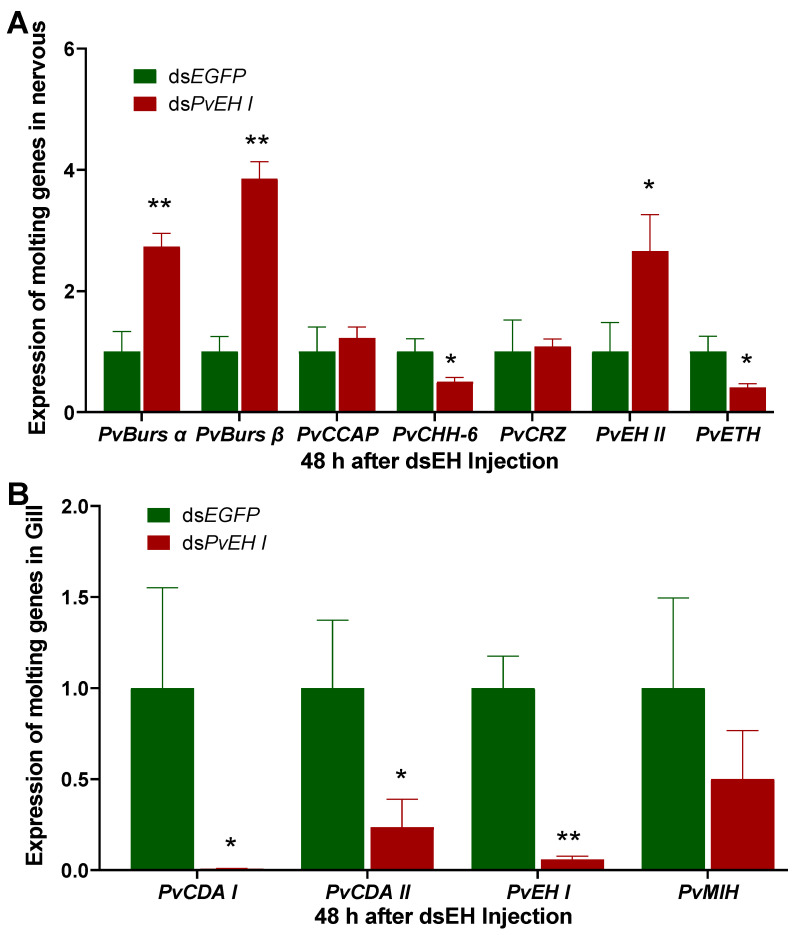
Effects of EH interference on the expression of molting-related genes in the nervous system (**A**) and gills (**B**). Due to the high expression of different molting-related genes in different parts of nervous systems, we use the whole nervous system as samples (including brain, eyestalk, circumesophageal ganglion, ventral nerve cord, etc.). * and ** represent significant differences and represent *p* < 0.05 or *p* < 0.01.

The impact of dsLvEH I injection on the expression of molting-related genes was invested, including MIH and molt-promoting genes such as CHH, chitin deacetylase (CDA), CRZ, ETH, crustacean cardioactive peptide (CCAP), Bursα, and Bursβ, which have been reported to play critical roles in the molting regulatory signaling pathways of other species (Figure 7). After *LvEH I* was silenced, the expression of its upstream genes *CHH-6*, *MIH,* and *CRZ* showed different transcription changes. *LvCHH-6* presented decreased expression (*p* < 0.05). However, *LvCRZ* and *LvMIH* did not show a significant transcription fluctuation (Figure 7). For the genes downstream of *LvEH I*, the transcription level of *LvBurs* (*LvBurs α* and *LvBurs β*) increased dramatically (*p* < 0.01), whereas *LvETH* presented significantly decreased expression (*p* < 0.05), and LvCCAP expression did not change significantly (Figure 7). In addition, another molt-related gene, *CDA,* was also tested, and the results revealed that both *LvCDA I* and *LvCDA II* presented significantly decreased transcription features (*p* < 0.05) (Figure 7B). Interestingly, the expression of *LvEH II* increased markedly (*p* < 0.05) after *LvEH I* was knocked down. In total, after *LvEH I* was silenced, *LvBurs* and *LvEH II* presented significantly increased transcription levels, whereas *LvCDA*, *LvCHH-6*, *LvETH,* and *LvMIH* presented decreased expression. *LvCCAP* and *LvCRZ* remained stable in terms of transcription (Figure 7).

## 3. Discussion

Given the economic significance of *L. vannamei* in aquaculture, the limited research on its molting physiology, and the pivotal role of EH in molting, this study employed molecular cloning, RT-qPCR, and RNAi techniques to investigate the regulatory function of EH in the molting process of *L. vannamei*. Through gene cloning, as well as structural and phylogenetic analyses, we identified and characterized two *EH* genes, *LvEH I* and *LvEH II*. *LvEH I* was prominently expressed in the gill, epidermis, and eyestalk, while *LvEH II* was more active in the eyestalk and brain. Notably, both genes showed stage-specific expression peaks, with *LvEH I* highly expressed during stages D1 (gill), D2 (eyestalk, epidermis, and gill), and D3 (epidermis and gill), whereas *LvEH II* was mainly expressed in D2 (brain) and D3 (eyestalk). After *LvEH I* was silenced, *LvCDAs*, *LvCHH-6*, *LvETH,* and *LvMIH* all presented decreased expression, *LvCCAP* and *LvCRZ* remained stable, and *LvBurs* and *LvEH II* presented significantly increased. In addition, the *LvEH I*-silenced groups presented significantly delayed molting advancement of *L. vannamei.*

Previous evidence in crustaceans suggests the presence of only a single ecdysis hormone (EH), as observed in *S. paramamosain* [24], *E. carinicauda* [21], and *L. vannamei* [25]. However, this study identified two distinct *EH*s in *L. vannamei*, highlighting the limited research on crustacean molting—a critical physiological process impacting production performance—and revealing the functional complexity of EH in molting regulation in this species. Evidence from insects also suggests the widespread existence of multiple EHs. For example, three *EH* genes were identified in both *Aedes aegypti* [26] and *Acyrthosiphon pisum* [27]. Additionally, the *T. castaneum* genome contains two *EH* genes, *EH* and *eclosion hormone-like* (*EHL*) [23], which have also been described as *EH1* and *EH2* [28]. *The LvEH I* and *LvEH II* proteins are structurally conserved. Both *LvEH I* and *LvEH II* contain the conserved domain of the eclosion superfamily, identifying them as members of the eclosion hormone. The eclosion superfamily is widely found in insects, and its members are considered key regulators of molting behavior. Additionally, the predicted mature peptides of *LvEH I* and *LvEH II* both include six cysteine residues and three corresponding disulfide bonds, which are crucial for protein stability and structure. These disulfide bonds may also contribute to functional regulation and protein interactions. Therefore, based on their structure, it is speculated that *LvEH I* and *LvEH II* play significant physiological roles in the molting behavior of *L. vannamei*.

In insects, *EH* is mainly produced by the glands of tracheales and neurosecretory cells on the ventral side of the brain [29,30,31] and is most highly expressed in the D stage of molting [32,33]. Crustaceans do not have tracheal glandular tissue, so the mechanism of action of EH in crustaceans is still unclear. In this study, the *LvEH I* gene presented the highest expression level in the gills of *L. vannamei,* followed by the epidermis, which is similar to the reports of *EH* in *E. carinicauda* [21]. The *LvEH II* gene was highly expressed in the nervous system of *L. vannamei*, similar to the expression pattern of *S. paramamosain* [24]. These results appear to support the presence of two EHs in crustaceans, suggesting that previous reports identifying only one EH might be due to technical limitations. In *Litopenaeus vannamei*, the expression levels of *LvEH I* and *LvEH II* were expressed at high levels in the D stage of molting (premolt stage) and presented relatively low expression levels in the A, B, and C stages, which is similar to reports of *EH* in *E. carinicauda* [21] and *S. paramamosain* [24]. In particular, *LvEHs* are highly expressed at D2 and D3 of the premolting period, which is a strong signal of the key role of LvEHs in the molting process of *L. vannamei*.

20E is currently recognized as a key regulatory signal for molting shared by crustaceans and insects, and EH is a key downstream molecule that responds to the 20E signal, and it synergizes with hormones such as CCAP, ETH, and Burs to regulate molting [20]. The molting-related functions of ETH, CCAP, and Burs are interconnected: ETH triggers the molting process and activates the secretion of CCAP, which facilitates the shedding of the old exoskeleton by coordinating muscle contractions. Meanwhile, Burs is responsible for the sclerotization and pigmentation of the newly formed exoskeleton [24,34,35]. The effects of *EH* deficiency on the expression of its downstream genes (*ETH*, *CCAP,* and *Burs*) have been reported in other species, and these genes show different fluctuation characteristics in different animals. For example, when red flour beetles were injected with ds*EHL*, the expression of *ETH*, *CCAP*, and *Bur* was significantly reduced. The transcription of the corresponding neuropeptide receptor genes (*ETHRA*, *ETHRB*, *CCAPR1*, *CCAPR2*, and *RK*) was also dramatically reduced [36]. Similarly, in *L. vannamei*, a doctoral dissertation reported that after *LvEH II* was silenced, its downstream genes (*ETH*, *ETHR*, *CCAP,* and *Burs*) all presented decreased expression levels [25]. Interestingly, mud crab molting-related genes presented different transcriptional changes. Specifically, after the injection of ds*EH* to juvenile crabs at stage D0, the transcription level of *ETH* increased sharply (+360%), but there was no change in the transcription level of *CCAP* [24]. These reports seem to indicate that the loss of *EH* does not necessarily cause the transcriptional downregulation of downstream pathway genes, which was also confirmed by our data. In this study, when *LvEH I* was knocked down, although *LvETH* presented decreased expression characteristics, the transcription level of *LvBurs* (*LvBurs α* and *LvBurs β*) increased dramatically, and CCAP expression did not change significantly. Notably, we found that *LvEH II* was significantly upregulated after *LvEH I* was silenced, which led us to speculate that there is some degree of gene redundancy between *LvEH I* and *LvEH II*. We also speculate that the upregulation of *LvEH II* therefore increased the expression of *LvBurs*. However, the upregulation of *LvEH II* cannot completely compensate for the delay in molting behavior caused by *LvEH I* deficiency, indicating that *LvEH I* is an indispensable critical factor for the normal occurrence of molting regulation in *L. vannamei*.

The secretion of 20E is also regulated by several key hormones upstream, including CHH, MIH, and CRZ. CHH and MIH inhibit the secretion of 20E in crustaceans [20,37], while CRZ promotes the secretion of 20E in crustaceans [38]. Because of the key roles of these hormones in the molting program of other species, we believe that focusing on their transcriptional changes will help to better understand the interactions of hormones. During molting, the upstream *CHH* and *MIH* molecules regulate the synthesis of ecdysone, thereby regulating the expression and secretion of *CRZ* and *EH* to initiate molting. In the premolting stage, *CRZ* initiates positive–negative feedback regulation of *EH* and *ETH* [23,38], and then, *EH* and *ETH* induce the expression of downstream functional genes to complete molting behavior [20,21,31,32,37,39,40,41]. The tacit coordination of these hormones is required for normal molting. In this study, when *LvEH I* was knocked down, *both LvCHH-6* and *LvMIH* presented decreased expression, whereas *LvCRZ* remained stable in terms of transcription. Our local transcriptome data confirm the prominent function of CDA in molting, which is also confirmed by studies in *Procambarus Clarkii* [42]. Both *LvCDA I* and *LvCDA II* also presented significantly decreased expression. This apparent transcriptional floating caused by *LvEH I* silencing suggests that these genes cooperate with *LvEH I* directly or indirectly. However, the specific regulatory network in *L. vannamei* remains to be further clarified.

The RNAi experiment in our study verified that *LvEH I* is necessary for the normal molting of *L. vannamei*. When *LvEH I* was knocked down, the molting process of *L. vannamei* was significantly delayed compared with that in the control groups, which is consistent with reports in insects and crustaceans. In insects, *EH* has been defined as one of the primary neuromodulators involved in the control of ecdysis, and its function has been examined in *Lepidoptera* and *Diptera* [43]. In *M. sexta*, the addition of *EH* can induce the ecdysis motor program of the isolated central nervous system (CNS) [33]. In *Drosophila*, Eileen Krüger [22] isolated an *EH* null mutant and used it to investigate the role of *EH* in larval ecdysis. The lack of *EH* function is completely lethal, which causes most *Drosophila* to die during the larval stages around the end of ecdysis. In the red flour beetle, both the classical *EH* gene orthologous group and the novel orthologous cluster of the *EHL* gene have been shown to be important for ecdysis. Knockdown of *EH* by RNAi resulted in the severe weakening of the preecdysis motor program and complete suppression of ecdysis [23]. When ds*TcEHL* was injected into pharate pupae, red flour beetles successfully molted into the adult stage, while approximately 80 % of adults died with untanned cuticles at 2 h post eclosion knockdown [36]. In *Leptinotarsa decemlineata*, the knockdown of *EH* at the final instar stage slightly impaired pupation and significantly affected wing expansion. Approximately 20% of the *LdEH* RNAi larvae remained as prepupae, which were completely wrapped in the old larval cuticles. These prepupae gradually darkened, dried, withered, and finally died. The remaining (approximately 80%) *LdEH* hypomorphs became pupae and emerged as abnormal adults, bearing smaller and wrinkled elytra and hindwings [44]. Compared with those in insects, there are relatively few reports of molting in crustaceans, and the physiological mechanisms of molting are largely unknown. In mud crabs, when juvenile crabs at the D0 stage were injected with ds*EH*, 58% of the crabs died before the D2 stage [24]. For *E. carinicauda*, the silencing of *EcEHL* via dsRNA delays both the molting process and the ecdysis rate [21]. Our data are consistent with those of many *EH*-related studies, which proves that *LvEH I* is essential for the normal occurrence of *L. vannamei* molting behavior and indicates that *LvEH I* is highly functionally conserved in molting regulation.

With respect to the omitted research on *LvEH II* knockdown and related hormone tracking in this study, during the course of our research, we retrieved a doctoral dissertation that systematically explored *LvEH II*’s role in *L. vannamei* molting [25]. The author reported that RNAi with *LvEH II* significantly delayed the molting of *L. vannamei* and obviously inhibited shrimp growth. Moreover, the injection of ds*LvEH II* into *L. vannamei* significantly inhibited the expression of the molting-related genes *LvETH*, *LvETHR*, *LvBurs,* and *LvCCAP* [25]. These results of *LvEH II* from that doctoral dissertation showed some similarities and some differences from the *LvEH I*-related research in our study. Together, we concluded that *LvEH I* and *LvEH II* are two different *EH* factors and that both play important roles in the molting program of *L. vannamei*. In addition, on the basis of the significantly different tissue distributions, different stages of high expression, and obviously different expression regulation of downstream pathway genes, we believe that the specific pathways by which *LvEH I* and *LvEH II* exert their molt-promoting functions are not identical.

## 4. Materials and Methods

### 4.1. Aquaculture Procedures and Sampling of Shrimp

*L. vannamei* fries were purchased from Hainan Blue Ocean Biotechnology Co., Ltd (Wenchang, China). and raised under consistent laboratory conditions (water temperature, 28 °C; salinity, 28‰; pH, 7.9 ± 0.5; dissolved oxygen > 5.0 mg/L; and ammonia nitrogen < 0.1 mg/L) until 3 months of age for the experiments.

Three healthy 3-month-old *L. vannamei* were anesthetized in an ice bath, and their tissues in cephalothorax were carefully dissected and promptly collected. The samples were then flash-frozen in liquid nitrogen, thoroughly ground into a fine powder, and stored at −80 °C for subsequent gene cloning analyses.

Three-month-old healthy *L. vannamei* (6.66 ± 0.5 cm in length and 4.5 ± 1 g in weight) were randomly selected and anesthetized in an ice bath. Quick dissection was conducted to collect 10 tissues, including epidermis, intestine, hepatopancreas, muscle, brain, gill, nerve, stomach, heart, and eyestalk. Three parallel samples were collected, each consisting of a mixture of the same tissue from five shrimp. The samples were immediately frozen in liquid nitrogen, fully ground into powder, and stored at −80 °C for LvEH tissue expression studies.

A microscope was used to observe morphological changes in the tail fin bristles of three-month-old healthy *L. vannamei*. In accordance with the different molting stages [9], samples from seven molting stages (A, B, C, and D0–D3) were collected. The shrimp were anesthetized on ice and quickly dissected. Tissues with high-level *EH* gene expression were sampled. *LvEH I* included gill, epidermis, and eyestalk. *LvEH II* included eyestalks and the brain. Three parallel samples were collected, each consisting of a mixture of the same tissue from five shrimp. The tissue samples were quickly frozen in liquid nitrogen, fully ground into powder, and stored at −80 °C for subsequent detection of *LvEHs*’ expression levels in different molting stages.

### 4.2. Molecular Cloning and Biological Analysis of LvEHs

Total RNA from the collected tissues in cephalothorax was extracted via the Animal Tissue Total RNA Extraction Kit (Foregene, Chengdu, China). The quality and concentration of the RNA were assessed via an Agilent 2100 Bioanalyzer (Agilent Technologies, Santa Clara, CA, USA) and a NanoDrop2000 spectrophotometer (Thermo Fisher Scientific, Waltham, MA, USA). The integrity of the total RNA was checked via 1.2% agarose gel electrophoresis. Qualified RNA was reverse transcribed to complementary DNA (cDNA) via the PrimeScript^TM^ RT reagent Kit with gDNA Eraser (Takara, Kyoto, Japan). Based on the transcriptome sequencing data of *L. vannamei*, the full-length CDSs of LvEHs were obtained from the NCBI database via BLAST. The primers used were designed via Primer Premier 5 software and NCBI Primer-BLAST (https://www.ncbi.nlm.nih.gov/tools/primer-blast/ (accessed on 14 June 2022)). Polymerase chain reaction (PCR) was performed with a 50 μL total volume that contained 25 μL of PrimeSTAR^®^ HS Premix ( Takara, Kyoto, Japan), 1 μg of cDNA, and 1 μL of forward or reverse primer, and ddH_2_O was added to 50 μL. The PCR program was as follows: 98 °C for 3 min; followed by 35 cycles at 98 °C for 10 s, 58 °C for 15 s, and 72 °C for 60 s; 72 °C incubation for 5 min; and then storage at 4 °C. The PCR products were purified and subcloned and inserted into the pEASY^®^-Blunt Zero Cloning Vector (TransGene Biotech, Beijing, China) for sequencing.

After the vector sequence was removed via Chromas2 software, the cDNA sequence was analyzed via DNA MAN 6 software for the prediction of amino acid translation start and stop codons. Sequence alignment and homology analysis were performed via the NCBI online program (https://blast.ncbi.nlm.nih.gov/Blast.cgi (accessed on 8 November 2022)). The online program Clustal Omega (https://www.ebi.ac.uk/Tools/msa/clustalo/ (accessed on 8 November 2022)) was used to perform multiple sequence alignment of amino acid sequences from different species, and the alignment results were edited and visualized via Jalview 2.11.2.3 software. Based on the target amino acid sequences of different species, phylogenetic analysis of LvEHs was performed using MEGA5.0 obeying the neighbor-joining algorithm (confidence: bootstrap = 1000).

The online programs ExPASy’s ProtParam and ProtScale (https://web.ExPASy.org (accessed on 16 November 2022)) were used to analyze the physicochemical properties and hydrophobicity of LvEHs. Signal peptide prediction was performed via the online program SignalP-6.0 (https://services.healthtech.dtu.dk/services/SignalP-6.0/ (accessed on 8 November 2022)). DeepTMHMM (https://dtu.biolib.com/app/DeepTMHMM/run (accessed on 16 November 2022)) was applied to predict the transmembrane structure of LvEHs. The cleavage sites of the precursor peptides were predicted via NeuroPred (http://stagbeetle.animal.uiuc.edu/cgi-bin/neuropred.py (accessed on 16 November 2022)). The conserved regions and special sites of LvEHs were predicted via the NCBI online program (https://www.ncbi.nlm.nih.gov/Structure/cdd/wrpsb.cgi (accessed on 16 November 2022)). WebLogo3 (http://weblogo.threeplusone.com/create.cgi (accessed on 16 November 2022)) was used to align and visualize the conserved region sequences. SWISS-MODEL (https://swissmodel.ExPASy.org (accessed on 16 November 2022)) was used to predict the tertiary structure of the protein. PEP-FOLD3.5 (https://mobyle.rpbs.univ-paris-diderot.fr/cgi-bin/portal.py#forms::PEP-FOLD3 (accessed on 16 November 2022)) was used to predict the tertiary structure of the mature peptide.

### 4.3. Synthesis of dsRNA and Knockdown of LvEHI

The DNA templates for dsRNA synthesis were amplified via PCR with cDNAs or the pEGFP-N1 plasmid via the primers listed in Table 1. The PCR products were purified, and the dsRNAs were synthesized via a T7 RNAi Transcription Kit (Vazyme, China) following the manufacturer’s protocol. The dsRNAs were purified via the magnetic bead purification method. The quantity of synthesized dsRNAs was measured using a NanoDrop ND-1000 spectrophotometer (Thermo Fisher Scientific, Waltham, MA, USA) and 1.5% agarose gel electrophoresis. The qualified dsRNAs were stored at −80 °C until use.

*L. vannamei* at the D0 molting stage were selected for RNAi experiments. The experimental group was injected with double-stranded *LvEHI* (ds*LvEHI*), and the negative control group was injected with double-stranded enhanced green fluorescent protein (ds*EGFP*). The injection dose was 2 μg per gram of body weight (ds*LvEH I* and ds*EGFP* diluted to 20 μL with 1 × PBS), and the mixture was injected via a 25 μL microsyringe. The injection site was the base point of the shrimp’s fifth leg. At the same time, a blank control group was set up (shrimp not injected with anything). Forty-eight hours after injection, 6 shrimp were randomly selected from each group, and their whole nervous system and gill tissues were separated. The tissue samples were quickly frozen in liquid nitrogen, fully ground into powder, and stored at −80 °C until use. The morphological changes in the tail fins were recorded by a camera.

### 4.4. RT–qPCR

As described in Section 2.1, total RNA was extracted from the tissues to be tested, the RNA quality was controlled, and the RNA was reverse transcribed into cDNA. RT–qPCR was used to detect *LvEH* expression in 10 tissues (reference genes were *18S rRNA*, *elongation factor 1-α* (*EF1-α*), and *β-actin*), *LvEH* expression in different molting stages of high-expression tissues (reference genes were *18S rRNA* and *EF1-α*), and the expression of other related genes in the molting regulation pathway after *LvEH1* was knocked down (reference genes were *18S rRNA* and *EF1-α*). The primers used for RT–qPCR are shown in Table 1. We verified the specificity of these primers by sequencing their PCR products. A temperature gradient from 55 °C to 65 °C was used to determine the optimal annealing temperature of each pair of primers. The standard curve was obtained based on a concentration gradient (8-fold dilution, 3 duplications). The amplification efficiency of each pair of primers was ensured at 90–100%. SYBR Green I RT–qPCR was performed in a volume of 10 μL, which contained 5 μL of ChamQ Universal SYBR qPCR Master Mix (Vazyme, China), 0.5 μL of cDNA, 0.5 μL of primer (Table 1), and 4 μL of ddH_2_O. The PCR procedures were 3 min at 95 °C, followed by 40 cycles (10 s at 95 °C, 10 s at X (X represents the gene-specific annealing temperature) °C and 10 s at 72 °C), 10 s at 95 °C, 60 s at 65 °C, and then a ramp-up of 2 °C per 1.0 s to 97 °C to generate a melting curve.

### 4.5. Statistical Analysis

The 2^−ΔΔCT^ method was used to analyze all of the RT–qPCR data to obtain the relative expression levels of the genes. For the expression level of *LvEHs* in 10 tissues, the expression level in the heart was used as the calibrator, and the reference genes *18S rRNA*, *β-actin,* and *EF1-α* were used to calculate the geometric mean [45]. For the *LvEHs* expression in different molting stages, the expression levels of the C-stage genes were used as calibrators, and *18S rRNA* and *EF1-α* were used as reference genes [45]. For the above two experiments, SPSS software 27 was used to perform one-way analysis (one-way NOVA). To calculate the expression levels of related genes after *LvEH I* knockdown, we used the expression levels in dsEGFP or PBS tissues as calibrators and used the *18S rRNA* and *EF1-α* genes as references [45]. SPSS software 27 was used to perform a two-tailed *t*-test on the expression levels of the target genes. The Duncan method was used for multiple comparisons. GraphPad Prism 9.5 (GraphPad Software Inc., San Diego, CA, USA) was used for visualization. “*” and “**” represent significant differences (*p* < 0.05) and extremely significant differences (*p* < 0.01), respectively. The values of each ordinate in the figures are expressed as the mean ± SEM.

## 5. Conclusions

In conclusion, this study identified two *LvEH* genes *(LvEH I* and *LvEH II*) involved in the molting process of *L. vannamei*. The sequences of LvEH I and LvEH II shared several similarities, including the presence of hydrophobic signal peptides, a conserved eclosion superfamily domain, six highly conserved cysteine residues, three corresponding disulfide bonds, and a cell-exterior localization. Interestingly, these two genes exhibited different tissue distribution patterns. *LvEH I* was highly expressed in the gill, epidermis, and eyestalk, while *LvEH II* was primarily expressed in the eyestalk and brain. Both genes showed stage-specific expression peaks: *LvEH I* was highly expressed during stages D1 (gill), D2 (eyestalk, epidermis, and gill), and D3 (epidermis and gill), whereas *LvEH II* was predominantly expressed during stages D2 (brain) and D3 (eyestalk). Notably, the molting process was significantly delayed when *LvEH I* was deficient. In *LvEH I* knockdown, *LvETH* expression decreased, while the transcription of *LvBurs* (*LvBurs α* and *LvBurs β*) increased dramatically, and *CCAP* expression remained largely unchanged. Additionally, the knockdown of *LvEH I* also affected the expression levels of molting-related genes, including *CHH*, *MIH*, and *CDA*. This finding contrasts with a previous report where silencing of *LvEH II* downregulated the expression of all downstream genes. Therefore, we conclude that both *LvEH I* and *LvEH II* are essential regulators of *L. vannamei* molting, though their specific functional mechanisms differ.

## Figures and Tables

**Table 1:** Primer sequences and functions used in this study.

Primer Name	Primer Sequence (5′–3′)	Applications
PvEH I-F	TCGTATCCCTTGTGAGCAGC	LvEH I cloning
PvEH I-R	CGCACCAATCTTTTCAGC	
PvEH II-F	GGGGAAACAGCACGCCTC	LvEH II cloning
PvEH II-R	CGTCTCGTTCGGGGCTGA	
PvEH I-qF	GCTGATGTACCACGACCACT	qRT–PCR/synthesized dsRNA
PvEH I-qR	AATGAGGTCCTGTGGGTTCG	qRT–PCR/synthesized dsRNA
PvEH II-qF	GCAGTGCAAGGAGATGTACG	qRT–PCR
PvEH II-qR	AGGAAGCGATTGAAGGTGGAG	qRT–PCR
β-actin-qF	CGAGAAATCGTTCGTGAC	qRT–PCR
β-actin-qR	GATGGAGTTGTAGGTGGTCT	qRT–PCR
18S rRNA-qF	TATACGCTAGTGGAGCTGGAA	qRT–PCR
18S rRNA-qR	GGGGAGGTAGTGACGAAAAAT	qRT–PCR
EF1-α-qF	TGGCTGTGAACAAGATGGAC	qRT–PCR
EF1-α-qR	AGATGGGGATGATTGGGACC	qRT–PCR
EGFP-F	GCAGTGCTTCAGCCGCTAC	synthesized dsRNA
EGFP-R	GCTTCTCGTTGGGGTCTTTG	synthesized dsRNA
T7-EGFP-F	TAATACGACTCACTATAGGGGCAGTGCTTCAGCCGCTAC	synthesized dsRNA
T7-EGFP-R	TAATACGACTCACTATAGGGGCTTCTCGTTGGGGTCTTTG	synthesized dsRNA
T7-PvEH I-F	TAATACGACTCACTATAGGGTCGTATCCCTTGTGAGCAGC	synthesized dsRNA
T7-PvEH I-R	TAATACGACTCACTATAGGGCGCACCAATCTTTTCAGCC	synthesized dsRNA
PvCDA I-F	CAACTCGTTCGAACCCTGGA	qRT–PCR
PvCDA I-R	ACTCGTTCTTGAGCCAAGGG	qRT–PCR
PvCDA II-F	TGGGGCTTCCTCTACGACT	qRT–PCR
PvCDA II-R	GACACTTGTGGGGCATACG	qRT–PCR
PvMIH-F	TTGAGAAGCTGCTGTCGTCC	qRT–PCR
PvMIH-R	GCGTAGCAGTTACTCTTGCAC	qRT–PCR
PvBurs α-F	GTCATATCCGGGCTGCAACT	qRT–PCR
PvBurs α-R	CCTGACTCCTGGCAACACAT	qRT–PCR
PvBurs β-F	CCCTCCGTCAACACTCCTTC	qRT–PCR
PvBurs β-R	CGAATTCCCGCACTTGAAGC	qRT–PCR
PvCCAP-F	TATTGTTGGCTGCCCATTCCC	qRT–PCR
PvCCAP-R	CTTCGGCGACGATGTGCTT	qRT–PCR
PvCHH-6-F	AAGATCGCCTTCGTCTCTGC	qRT–PCR
PvCHH-6-R	CGTCGAAGACCTGCCTCTTT	qRT–PCR
PvCRZ-F	CTCCACCAGAACGCTGCTTA	qRT–PCR
PvCRZ-R	GTCGCCACCAGAGGAAAGAT	qRT–PCR
PvETH-F	GTTCCACCTGGAAGCGCGA	qRT–PCR
PvETH-R	GTCTCGGCGAAGAAATGCCC	qRT–PCR

## Data Availability

Data is contained within the article.
